# Latent profiles of post-traumatic distress and growth in adolescents: the role of modifiable protective factors

**DOI:** 10.3389/fpsyt.2026.1720487

**Published:** 2026-01-28

**Authors:** Haiying Yang, Lihong Sun, Ying Zhang

**Affiliations:** 1 School of Physical Education, Southwest Jiaotong University, Chengdu, China; 2 School of Physical Education, Hubei Second Normal University, Wuhan, China; 3 School of Education, Hubei Second Normal University, Wuhan, China

**Keywords:** adolescents, COVID-19, latent profile analysis, posttraumatic distress, posttraumatic growth

## Abstract

**Objective:**

This study examined heterogeneous patterns of trauma-related adaptation among Chinese adolescents during the post–COVID-19 recovery phase, focusing on the co-occurrence of posttraumatic distress (PTD) and posttraumatic growth (PTG). We also investigated how modifiable psychosocial protective and vulnerability factors were associated with membership in different adaptation profiles.

**Method:**

A large-scale cross-sectional survey was administered to 5, 044 students (aged 9–17 years; 46.6% male) from 15 primary and secondary schools in Wuhan, China. Validated instruments assessed posttraumatic stress symptoms (PCL-C), posttraumatic growth (PTGI), depressive symptoms (CES-D), and anxiety (SAS). Protective and vulnerability factors included resilience (CD-RISC), perceived social support (SSRS), physical activity (PARS-3), school belonging (PSSM), adaptive coping (SCSQ), and trait anxiety (TAI). Latent profile analysis (LPA) was used to identify adaptation profiles, and multinomial logistic regression examined how modifiable psychosocial factors were associated with profile membership.

**Results:**

LPA revealed four empirically derived profiles: a High Distress/High Growth–Moderate PTSD profile (76.9%), a Low Distress–High Growth profile (4.8%), a Low Growth–Moderate Distress profile (3.9%), and a High Distress/High Growth–High PTSD profile (14.4%). The vast majority of adolescents showed some degree of both PTD and PTG, consistent with dual-process perspectives. In multinomial models, higher resilience, social support, school belonging, adaptive coping, and physical activity were associated with greater likelihood of belonging to the Low Distress–High Growth profile rather than more distressed profiles, whereas higher trait anxiety was associated with increased odds of membership in profiles characterized by greater distress.

**Conclusions:**

In this large school-based sample of Chinese adolescents, distress and growth frequently co-occurred and clustered into distinct adaptation profiles that differed systematically in psychosocial resources. Resilience, social connectedness, school belonging, and physical activity emerged as promising targets for trauma-informed, school-based support, whereas trait anxiety appeared to mark heightened vulnerability. Given the cross-sectional and single-region design, these findings should be interpreted as exploratory, and longitudinal and cross-cultural studies are needed to clarify temporal and contextual influences on adolescent trauma adaptation.

## 1 Introduction

Adolescence constitutes a critical neurodevelopmental period characterized by rapid biological maturation, identity consolidation, and heightened vulnerability to environmental stressors. Trauma exposure during this sensitive phase can precipitate heterogeneous outcomes, spanning debilitating posttraumatic distress (PTD) to resilient adaptation and posttraumatic growth (PTG) (1, 2). Such variability challenges dichotomous conceptualizations of trauma responses, necessitating multidimensional models to elucidate the complexity of adolescent adaptation trajectories (3).

The COVID-19 pandemic, as a global collective trauma, has profoundly disrupted adolescent development through educational discontinuities, protracted social isolation, and familial stress escalation (4, 5). Paradoxically, emerging evidence highlights concurrent positive psychological transformations, including enhanced life appreciation and personal strength (6, 7). This coexistence of distress and growth underscores the limitations of unidimensional frameworks and calls for integrative models that capture the dialectical nature of trauma adaptation.

Contemporary theoretical advances posit PTD and PTG as concurrent, non-mutually exclusive outcomes mediated by dual cognitive processes: intrusive and deliberate rumination (8, 9). Intrusive rumination—marked by involuntary, distressing trauma-related cognitions—disrupts pre-existing schemas, compelling cognitive reappraisal (10, 11). Conversely, deliberate rumination facilitates constructive meaning-making through purposeful reflection, fostering PTG (12, 13). Critically, these processes dynamically interact; initial intrusive rumination may gradually give way to deliberate processing, enabling growth amidst distress (14).

Most existing trauma-adjustment research, primarily conducted in Western contexts, has utilized variable-centered analyses and trajectory modeling rather than person-centered profiling. This literature often distinguishes three broad patterns of response: a distress-dominant pattern (high PTD/low PTG), a resilient pattern (moderate PTD/PTG), and a growth-dominant pattern (low PTD/high PTG) (15, 16). Importantly, these studies were not designed to test a specific three-profile latent structure, and their conclusions may not generalize to collectivist cultural settings that emphasize interdependence, relational harmony, and different expressive norms for distress (17). Indeed, emerging evidence from Chinese populations reveals distinct adaptation patterns, with salient subgroups exhibiting concurrent high PTD and PTG—a profile relatively underrepresented in Western literature (18, 19). These findings suggest that collectivist contexts, where familial support and communal resilience are paramount, may foster unique trauma responses wherein growth can be facilitated despite persistent distress (20).

The present study addresses these gaps by employing Latent Profile Analysis (LPA) to: (1) identify culturally specific trauma adaptation profiles among Chinese adolescents in the post-pandemic context, (2) examine the differential predictive effects of key modifiable protective factors (e.g., resilience, social support, physical activity) and vulnerability factors (e.g., trait anxiety) on profile membership, and (3) evaluate a nested protective-factor model that integrates collectivist-oriented resources with individual-level resilience mechanisms. Given the limited person-centered evidence among adolescents in collectivist contexts, our use of LPA was primarily exploratory regarding the exact number and nature of profiles. Drawing on prior findings that PTD and PTG can co-occur (18, 19), we anticipated that, in addition to profiles broadly characterized by distress-dominant, resilient, and growth-dominant patterns, a distinct subgroup combining elevated PTD with moderate-to-high PTG might emerge. We further expected that collectivist-oriented resources (e.g., social support, school belonging) would show stronger associations with profiles characterized by relatively higher PTG.

Conceptually, we defined trauma adaptation as a higher-order construct characterized by the joint patterning of PTD and PTG. PTD was operationalized by three core symptom domains—posttraumatic stress, depressive symptoms, and anxiety—whereas PTG captured perceived positive psychological changes following trauma exposure. These four psychological indicators served as the manifest variables used to derive latent adaptation profiles. In contrast, coping styles, resilience, school belonging, social support, physical activity, and trait anxiety were treated as modifiable psychosocial factors that were examined as correlates of profile membership rather than as confirmed causal predictors.

Accumulating evidence suggests that several modifiable psychosocial factors confer protection against trauma-related psychopathology while promoting PTG. Higher resilience and perceived social support have consistently been linked with greater PTG and lower distress following diverse traumatic events (21–24). School belonging, as a key marker of institutional integration, buffers the adverse effects of adversity on mental health (25, 26). Similarly, engagement in regular physical activity has been associated with enhanced psychological well-being among Chinese adolescents during and after the COVID-19 pandemic (27, 28). Furthermore, adaptive coping strategies predict more favorable post-trauma adjustment, whereas elevated trait anxiety functions as a vulnerability factor that heightens the risk for persistent distress (29, 30). By elucidating how these factors differentiate distinct adaptation pathways, our findings aim to inform culturally congruent interventions that leverage indigenous protective systems, recognizing that the identified latent profiles reflect concurrent configurations of distress and growth rather than developmental stages or causal pathways, thereby promoting sustainable mental health outcomes for adolescents in collectivist societies.

## 2 Methods

### 2.1 Participants

In this cross-sectional study, participants were recruited using a school-based convenience sampling approach from 15 public primary and secondary schools located in different urban districts of Wuhan, China, in order to increase demographic diversity in the sample. Prior to data collection, permission was obtained from school administrators, and informed consent was secured from both the students’ guardians and the students themselves. The study was reviewed and approved by the Institutional Ethics Committee. The inclusion criteria for participants were as follows: (a) students aged between 9 and 17 years; (b) no known diagnosis of psychiatric disorders; (c) no history of use of barbiturates, benzodiazepines, or chloral hydrate; and (d) no contraindications to physical activity. A total of 5, 044 students met the criteria and voluntarily participated in the study.

The data analysed in the present study were originally collected in October 2021 as part of a routine, school-based mental health screening organized by the local education authorities. At the time of data collection, all procedures followed national guidelines for school psychological assessment, and written informed consent was obtained from students and their legal guardians. The current secondary analysis of the anonymized dataset was subsequently reviewed and approved by the Ethical approval was granted by the Medical Ethics Committee of Southwest Jiaotong University in 2022 (Approval No: SWJTU-2201-SK (002)). All procedures were conducted in accordance with the Declaration of Helsinki.

### 2.2 Procedures

Data were collected in October 2021, almost two years after the initial COVID-19 outbreak in Wuhan, China (December 2019–January 2020). Data collection was conducted in classroom settings during regular school hours across participating primary schools in Wuhan. Prior to survey administration, researchers obtained permission from school authorities and distributed informed consent forms to both students and their guardians. The timing of data collection was strategically chosen to capture adolescents’ psychological responses approximately 10 months after the initial COVID-19 outbreak and lockdown in Wuhan, allowing sufficient time for both immediate and delayed trauma responses to manifest (31).

The assessment comprised self-report questionnaires delivered in paper format: a general demographic survey, a psychological adaptation battery (including CESD, PTGI, PTSD, and SAS), and a series of validated instruments measuring modifiable protective factors (e.g., resilience, physical activity, coping strategies, social support, and school belonging). Each assessment session lasted approximately 30 minutes.

To ensure consistency and data quality, trained research assistants administered the survey in classroom settings, providing standardized instructions and clarifying any questions. Research assistants received 2 hours of specialized training on trauma-informed data collection practices and were supervised by licensed psychologists throughout the data collection process. To enhance data quality and minimize missing responses, all questionnaires were checked immediately after completion. In cases of item omission or logical inconsistencies, participants were asked to review and correct their answers on site. The entire research team underwent unified training prior to field implementation to ensure procedural consistency and ethical compliance across all schools involved.

### 2.3 Measures

#### 2.3.1 General information questionnaire

Participants completed a demographic survey that captured key background information, including gender, age, household registration (urban/rural), only-child status, family structure (two-parent vs. single-parent household), family relationship quality, and parents’ educational attainment.

#### 2.3.2 Psychological adaptation indicators

Consistent with our conceptualization of trauma adaptation, four established self-report scales were used to index PTD and PTG. PTD was reflected by three symptom domains: depressive symptoms (CES-D), PTSD symptoms (PCL-C), and anxiety (SAS), while PTG was assessed directly using the Posttraumatic Growth Inventory (PTGI). Standardized scores on these four measures served as manifest indicators in the LPA to identify distinct profiles of trauma adaptation.

CES-D (Center for Epidemiologic Studies Depression Scale): A 20-item instrument assessing depressive symptoms experienced in the past week. Each item is rated on a 4-point Likert scale ranging from 0 (“rarely or none of the time”) to 3 (“most or all of the time”). Higher scores are indicative of greater depressive symptoms. The CES-D has been extensively validated across different cultural settings, including Chinese populations, and has demonstrated strong reliability (α=0.89) in youth samples (32).

PTGI (Posttraumatic Growth Inventory). The PTGI, developed by Tedeschi and Calhoun, is a 21-item scale that assesses perceived positive psychological changes following trauma (33). Respondents rate the degree to which they experienced each change on a 6-point Likert scale ranging from 0 (“no change”) to 5 (“very great degree of change”), with higher scores indicating greater posttraumatic growth. The Chinese version has demonstrated good reliability and validity in adolescent samples (34). Internal consistency estimates (Cronbach’s α) for the PTGI and all other measures and subscales in the present sample are reported in the **Supplementary Materials (Table S)**.

PTSD Checklist-Civilian Version (PCL-C): The PCL-C, a 17-item scale developed by Weathers (35), is widely used to assess PTSD symptoms in civilian populations. It measures three key symptom clusters: intrusion, avoidance, and hyperarousal. Responses are based on a 5-point Likert scale, ranging from “not at all” to “extremely”. The scale is well-validated across diverse populations, including Chinese youth, with a reported Cronbach’s alpha of 0.91 (36).

SAS (Self-Rating Anxiety Scale): The SAS, developed by Zung (37), is a 20-item self-report measure of anxiety. It assesses both cognitive and somatic symptoms of anxiety, with each item rated on a 4-point scale. The Chinese version of the SAS has been validated in adolescent samples, with strong psychometric properties (Cronbach’s α=0.88) (38). In subsequent analyses, higher scores on the CES-D, PCL-C, and SAS were interpreted as higher levels of posttraumatic distress, whereas higher PTGI scores reflected greater PTG.

#### 2.3.3 Modifiable psychosocial protective and vulnerability factors

We assessed a set of modifiable psychosocial and environmental factors that were conceptualized either as protective (i.e., resources that promote adaptive outcomes) or as vulnerability indicators (i.e., factors that increase the likelihood of distress). Protective factors included resilience, physical activity, perceived school belonging, perceived social support, and positive coping. Negative coping and trait anxiety were treated as vulnerability indicators. For brevity, we refer to this set collectively as “modifiable psychosocial factors”.

CD-RISC (Connor-Davidson Resilience Scale): The CD-RISC is a 25-item scale developed by Connor and Davidson (39) to assess psychological resilience. It measures individuals’ capacity to cope with adversity and to maintain psychological well-being in stressful situations. Participants rate each item on a 5-point Likert scale from “not true at all” to “true nearly all the time.” The scale has shown high reliability (α=0.94) in adolescent populations in China (40).

PARS-3 (Physical Activity Rating Scale): PARS-3 is a validated instrument for comprehensive assessment of physical activity engagement. The scale quantifies three key dimensions: (1) exercise intensity (rated 1-5), (2) duration (scored 1-5), and (3) frequency (recorded weekly occurrences). The composite physical activity score is derived through the formula: intensity × duration × frequency, yielding a continuous metric ranging from 0 to 100. Based on established cutoffs (41), scores are categorized into: low (≤19), moderate (20-42), and high (≥43) activity levels. The measure has demonstrated excellent psychometric properties in Chinese adolescent populations, with a Cronbach’s α of 0.80 (27, 28), supporting its reliability for mental health research applications.

PSSM (Psychological Sense of School Membership): The PSSM is an 18-item self-report instrument assessing students’ perceived belongingness within the school environment. Utilizing a 5-point Likert scale (1=“strongly disagree” to 5=“strongly agree”), the PSSM evaluates affective and cognitive components of school connectedness. The measure has been extensively validated cross-culturally, with Chinese adolescent samples demonstrating exceptional internal consistency (α=0.93; 42). Its strong psychometric properties have established the PSSM as a gold-standard measure in educational psychology research.

SCSQ (Simplified Coping Style Questionnaire): Developed by Xie (43) and grounded in transactional model of stress and coping, the SCSQ is a 20-item instrument assessing adaptive and maladaptive coping strategies. The SCSQ yields two distinct subscales: (1) positive coping (12 items) and (2) negative coping (8 items). Recent validation studies with Chinese adolescent populations have confirmed the measure’s robust reliability (Cronbach’s α=0.83; 44), supporting its utility for examining stress response patterns in developmental samples.

SSRS (Social Support Rating Scale): The SSRS, developed by Xiao (45), measures perceived social support from three sources: family, friends, and others. It includes 10 items and assesses both subjective and objective aspects of social support. This scale is widely used in Chinese psychological research and has shown excellent internal consistency (α=0.91) in adolescent samples (46).

TAI (Trait Anxiety Inventory): Trait anxiety was assessed using the TAI, the trait subscale of the State–Trait Anxiety Inventory (STAI; 47). The TAI consists of 20 items rated on a 4-point Likert scale ranging from 1 (“almost never”) to 4 (“almost always”), with higher total scores indicating greater dispositional anxiety. The Chinese version of the TAI has demonstrated good reliability and validity in adolescent samples, and in the present study trait anxiety was conceptualized as a vulnerability factor and used as a predictor of latent profile membership in regression models rather than as a manifest indicator in the LPA.

In the present study, the positive coping subscale was used as an index of adaptive coping (protective factor), whereas the negative coping subscale was used as an index of maladaptive coping (vulnerability factor) in regression analyses; higher scores indicate more frequent use of the respective coping style. For consistency and clarity, Cronbach’s α coefficients for all instruments and subscales used in the present analyses (CES-D, PTGI, PCL-C, SAS, CD-RISC, PARS-3, PSSM, SCSQ positive and negative coping subscales, SSRS, and TAI) were calculated in this sample and are summarized in the **Supplementary Materials (Table S)**.

### 2.4 Data analysis

All statistical analyses were conducted using SAS 9.4 (version 9.4, SAS Institute, Inc., Cary, NC, USA) and R software (version 4.3.4) for data cleaning and analysis. Missing values for all variables in this study were less than 1%, and mean imputation was used to fill in missing quantitative data (48).

Descriptive analyses were performed following rigorous data screening procedures. Normality of continuous variables was systematically evaluated using the Shapiro-Wilk test. Parametric data were presented as mean ± standard deviation (SD), while non-normally distributed variables were reported as median with interquartile range (IQR; 25th-75th percentiles). Categorical variables were summarized using frequency counts and percentages (n, %). All inferential tests were conducted as two-tailed analyses with an alpha level of 0.05 defining statistical significance. When comparing mean scores across the four latent profiles, we used Kruskal–Wallis tests for core psychological outcomes (CESD, PTGI, PTSD, and SAS) that violated normality assumptions and one-way ANOVA for normally distributed psychosocial predictors. Significant omnibus effects were followed by pairwise *post hoc* comparisons (Dunn–Bonferroni tests after Kruskal–Wallis; Tukey’s HSD after ANOVA) to examine differences between each pair of latent classes.

LPA was conducted using the tidyLPA package to identify latent subgroups based on participants’ responses to core psychological indicators. Models specifying two to five latent classes were estimated and compared using multiple information criteria: Akaike Information Criterion (AIC), Bayesian Information Criterion (BIC), sample-size adjusted BIC (aBIC), and entropy. Lower values of AIC, BIC, and aBIC indicate better model fit, while entropy values range from 0 to 1; values >0.80 are typically taken to indicate high classification precision, in that most individuals are assigned to their most likely latent class with average posterior membership probabilities exceeding 0.90 (49). The Lo-Mendell-Rubin adjusted likelihood ratio test (LMR-LRT) and Bootstrap Likelihood Ratio Test (BLRT) were used for model comparison, with significant p-values (p < 0.05) indicating that the k-class model fits significantly better than the (k-1)-class model (50). Additionally, the interpretability of profiles and theoretical relevance were considered when selecting the final model.

In multivariable analysis, multinomial logistic regression models were employed to explore the effects of different scales on children in different latent groups, treating class membership as the categorical outcome variable. Multicollinearity diagnostics were performed prior to model estimation; predictors with variance inflation factors (VIF) exceeding 10 were excluded to ensure model stability (51). Binary logistic regression was further conducted to analyze influencing factors for membership in the cluster group of particular interest (high-risk group as identified by LPA). Odds ratios (ORs) and 95% confidence intervals (CIs) were reported for all regression models. All statistical procedures were conducted following established guidelines for latent class analysis and multinomial regression (52).

## 3 Result

### 3.1 LPA

#### 3.1.1 Latent profile model selection and fit indices

To determine the optimal number of latent profiles representing psychological adaptation patterns among adolescents, an LPA was conducted.

Model fit statistics for two- through five-class solutions are summarized in **Table 1**. Although the 2-class solution exhibited high entropy (0.990) and a significant BLRT (p=.010), over 92% of participants clustered in one profile, indicating insufficient differentiation. The 3-class model improved fit (AIC=49 193; BIC=49 310) and entropy (0.996), but its class proportions (77.68%, 14.35%, 8.25%) offered only marginal gains in practical interpretability.

**Table 1:** Model fit indices for LPA models with 2 to 5 classes.

Classes	Percent (%)	LogLik	AIC	BIC	aBIC	Entropy	BLRT
2	92.01/7.99	-26075	52177	52261	52220	0.990	0.010
3	77.68/14.35/8.25	-24578	49193	49310	49253	0.996	0.010
4	76.88/4.80/3.93/14.39	-23359	46764	46914	46841	0.994	0.010
5	17.15/60.03/0.14/14.39/8.298	-24579	49214	49396	49307	0.489	0.960

Entropy values closer to 1 indicate better classification accuracy. AIC, BIC, and aBIC are penalized likelihood criteria, with lower values indicating better fit. BLRT compares model k vs. k-1; a significant p-value (<.05) favors model k. Class proportions represent the percentage of individuals assigned to each latent profile.

The 4-class solution struck the best balance between statistical indicators and theoretical meaningfulness. It achieved the lowest AIC (46 764), BIC (46 914), and aBIC (46 841), maintained high entropy (0.994), and produced a significant BLRT (p=.010). Moreover, its four profiles—comprising 76.88%, 4.80%, 3.93%, and 14.39% of the sample—provided a theoretically interpretable solution; however, the two smaller classes (4.80% and 3.93%) represent relatively small subgroups, which may reduce profile stability and replicability. Accordingly, results involving these smaller profiles should be interpreted cautiously and verified in independent samples.

**Figure 1:**
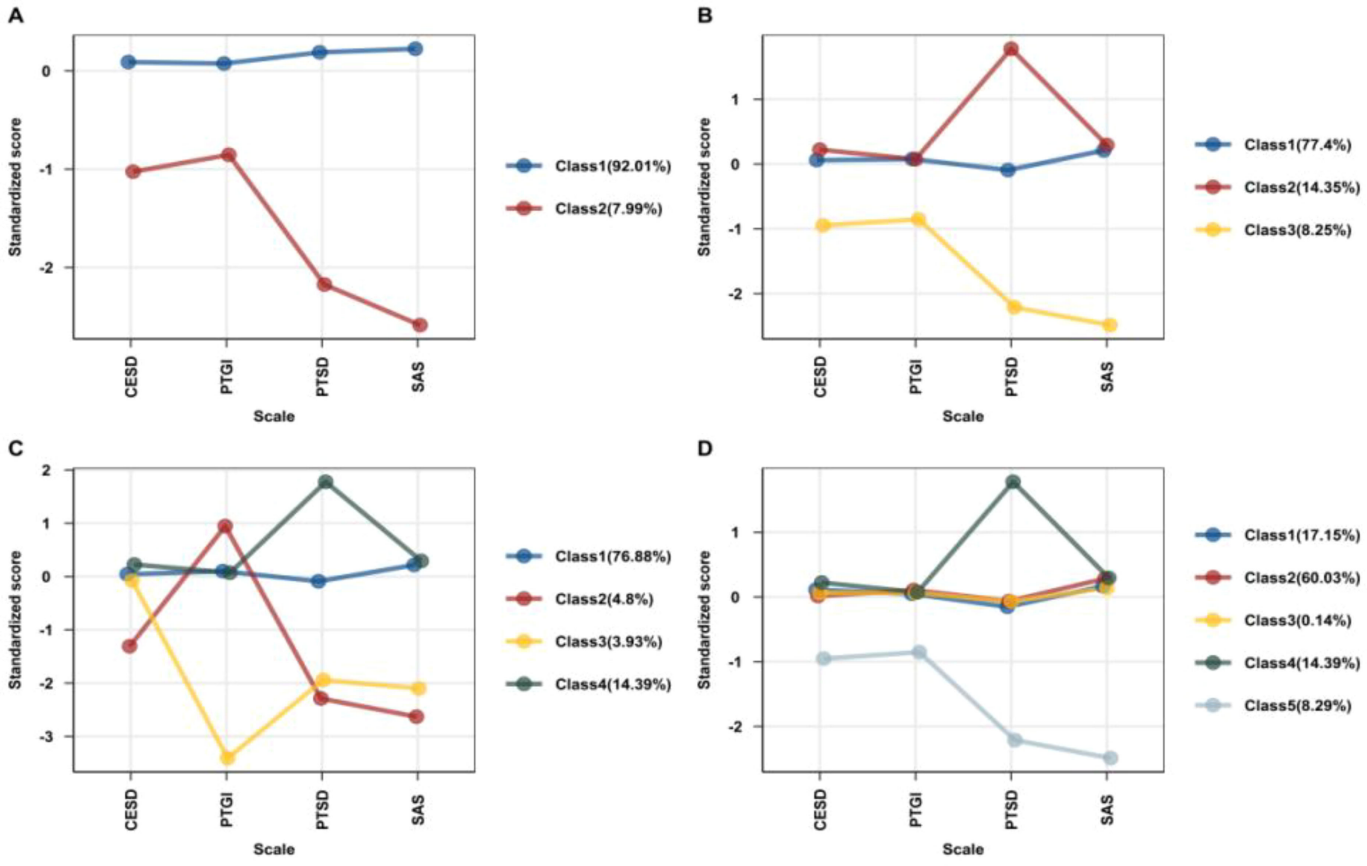
Standardized factor scores for each latent profile solution **(A)** displays the two-class solution, **(B)** illustrates the three-class solution, **(C)** presents the four-class solution, and **(D)** shows the five-class solution. CESD, Center for Epidemiologic Studies Depression Scale; PTGI, Posttraumatic Growth Inventory; PTSD, Posttraumatic Stress Disorder; SAS, Self-Rating Anxiety Scale.

By contrast, the 5-class model evidenced overfitting: entropy dropped sharply to 0.489, information criteria increased, and BLRT became non-significant (p=.960), signaling poor classification reliability. Following best practices, we therefore selected the 4-class model as the optimal solution for subsequent analyses. **Figure 1** illustrates the standardized scores of participants across four key psychological indicators- CESD, PTGI, PTSD, and a SAS-within the latent classes identified in the 2-class, 3-class, 4-class, and 5-class LPA models. Comparing the 2–5 class solutions, the 4-class model offered the optimal balance of statistical fit and interpretability.

This solution identified four distinct profiles whose labels emphasize their most distinguishing features in posttraumatic distress and growth. Class 1 (76.9%) was labelled High Distress/High Growth–Moderate PTSD, characterized by high PTG alongside moderate-to-high anxiety and depression and moderately elevated PTSD symptoms. Class 2 (4.8%) was labelled Low Distress–High Growth, exhibiting the highest PTG with the lowest distress levels across all indicators. Class 3 (3.9%) was labelled Low Growth–Moderate Distress, showing the lowest PTG with moderate-low levels of PTSD, anxiety, and depression. Finally, Class 4 (14.4%) was labelled High Distress/High Growth–High PTSD, displaying similarly high anxiety and depression as Class 1 but the highest PTSD levels combined with moderate-high PTG. Thus, the 4-class solution most clearly delineates distinct post-trauma adaptation pathways among adolescents, including two mixed distress–growth profiles that differ primarily in PTSD severity.

#### 3.1.2 Latent class characteristics

**Figure 2:**
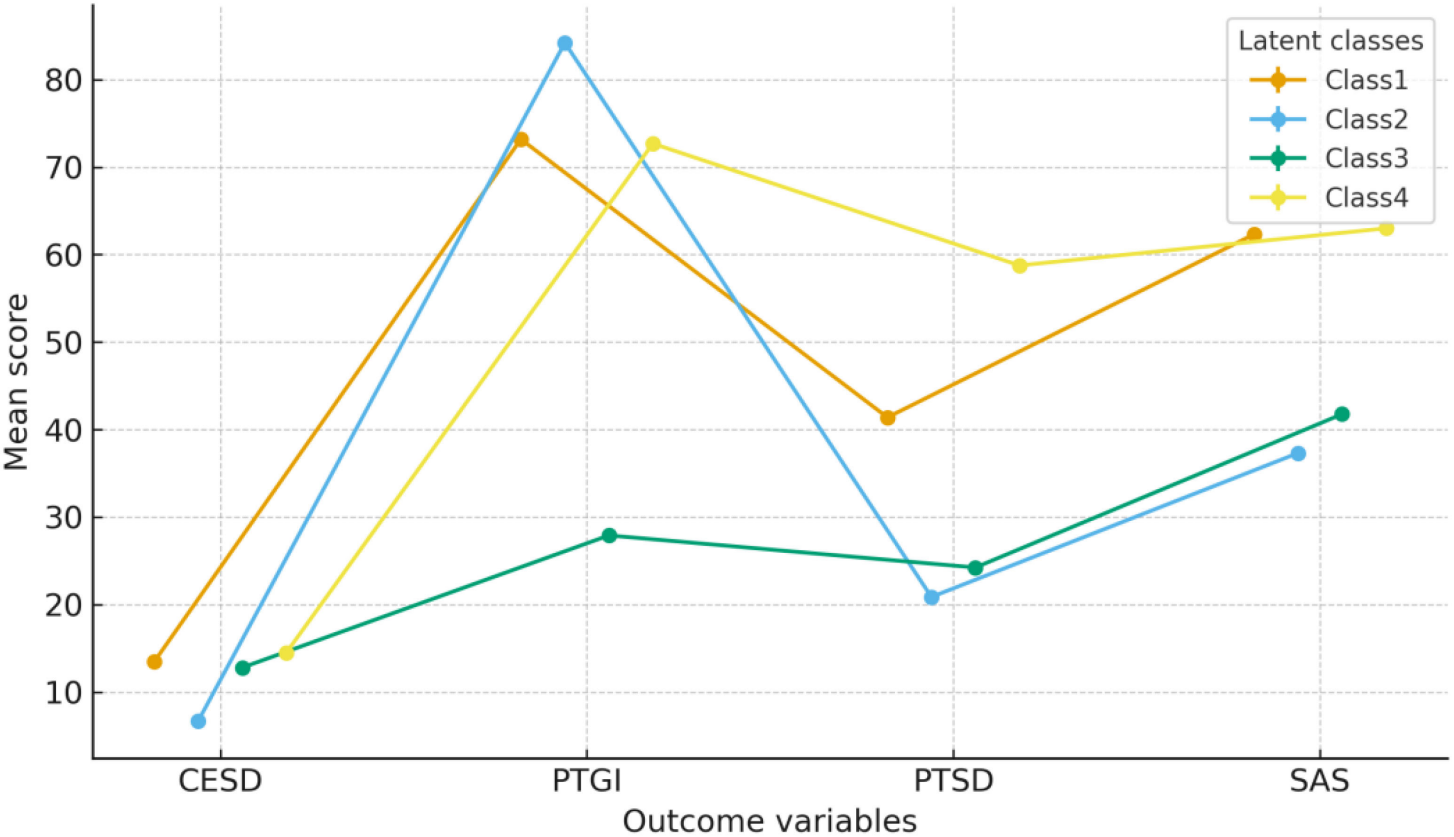
Mean levels ( ± SE) of depressive symptoms, posttraumatic growth, PTSD symptoms, and anxiety across the four latent profiles.

As some of the psychological outcome variables (CESD, PTGI, PTSD, and SAS) did not meet the assumptions of normality, we conducted non-parametric Kruskal–Wallis tests to examine group differences across the latent classes (53), reporting H statistics for each comparison. The four latent classes differed significantly on all psychological outcomes (H=343.32–2709.19, p<.001). **Figure 2** displays the mean ± standard errors for CESD, PTGI, PTSD, and SAS across the four profiles; the corresponding descriptive statistics and Dunn–Bonferroni *post hoc* pairwise comparisons are provided in **Supplementary Table S2**.

The *High Distress/High Growth–Moderate PTSD* profile (Class 1; n=3, 878) showed high PTG (73.19 ± 7.64) co-occurring with moderate-to-high levels of PTSD (41.39 ± 3.32) and anxiety (62.32 ± 5.55). The *Low Distress–High Growth* profile (Class 2; n=242) displayed the highest PTG (84.21 ± 5.73) and the lowest scores on all distress indicators, including PTSD (20.86 ± 3.85) and depression (6.71 ± 6.08). The *Low Growth–Moderate Distress* profile (Class 3; n=198) was marked by the lowest PTG (27.90 ± 7.32) alongside moderate-low distress. Finally, the *High Distress/High Growth–High PTSD* profile (Class 4; n=726) reported the highest levels of PTSD (58.76 ± 2.96), depression (14.49 ± 6.50), and anxiety (63.00 ± 5.98), while simultaneously reporting moderate-high PTG (72.69 ± 9.53).

#### 3.1.3 Group differences in modifiable protective factors across latent profiles

**Figure 3:**
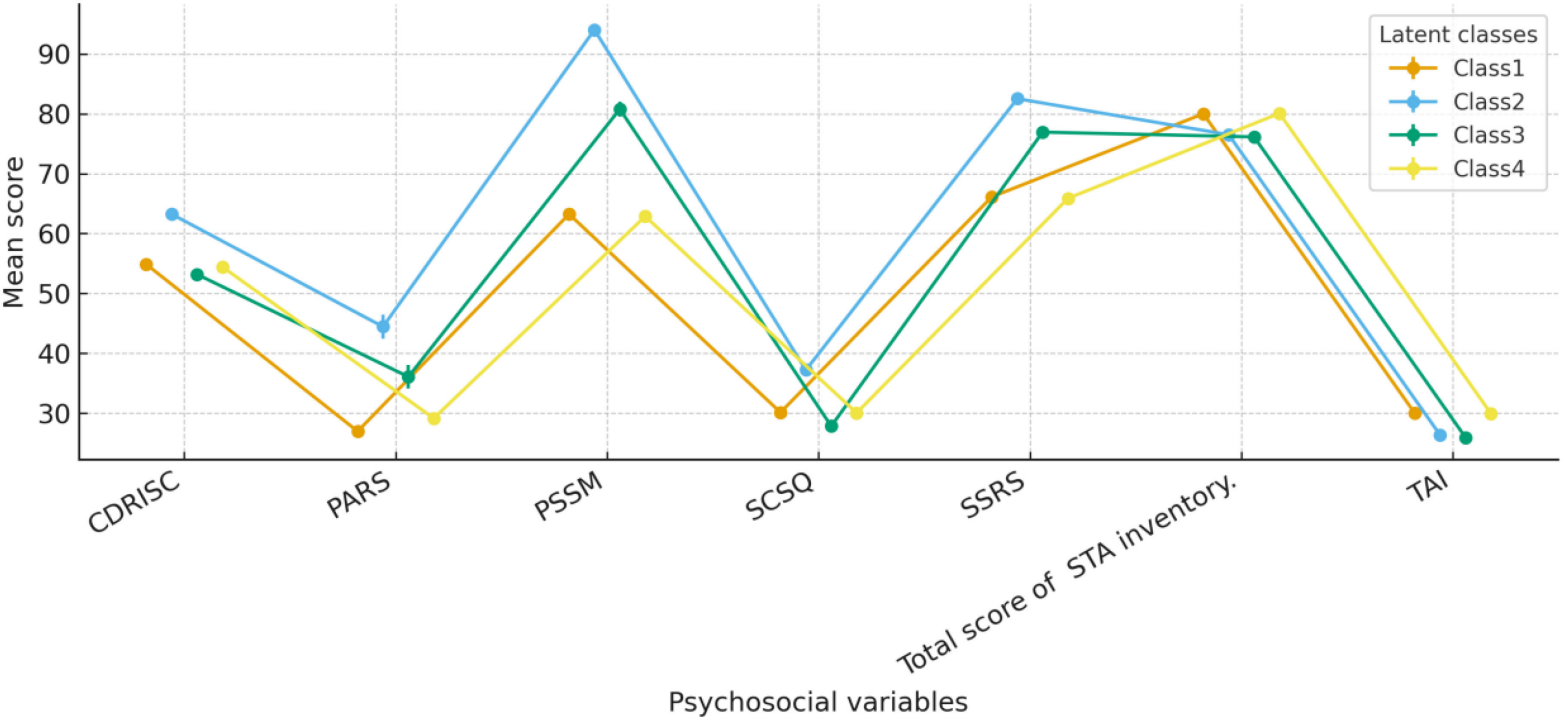
Mean levels ( ± SE) of resilience, physical activity, school belonging, social support, adaptive coping, state anxiety, and trait anxiety across the four latent profiles.

One-way ANOVAs revealed significant omnibus differences among the four latent classes on all seven psychosocial variables—five protective indicators (resilience, physical activity, school belonging, social support, and adaptive coping) and two vulnerability indicators (state and trait anxiety; all F’s > 50.71, p <.001). **Figure 3** shows the mean levels (± standard errors) of these psychosocial factors across the four profiles. As summarized in **Figure 3** and detailed in **Supplementary Table S3**, adolescents in the Low Distress–High Growth profile (Class 2) exhibited the most favourable pattern (highest resilience, physical activity, school belonging, social support, and adaptive coping, and the lowest trait anxiety), whereas the Low Growth–Moderate Distress profile (Class 3) reported the lowest levels of resilience and coping. The two mixed profiles (Classes 1 and 4) showed intermediate levels of most protective factors but relatively elevated state and trait anxiety. Given the overlap with the multivariate results presented below, these descriptive findings are reported briefly in the main text.

#### 3.1.4 Multinomial logistic regression: psychosocial correlates of latent profile membership

To examine how modifiable psychosocial factors differentiated the four latent adaptation profiles, we estimated a multinomial logistic regression model with latent class membership as the outcome. The High Distress/High Growth–Moderate PTSD profile (Class 1) was specified as the reference category, consistent with the presentation in **Table 2**. This approach allowed us to contrast the three remaining profiles against the most prevalent mixed distress–growth pattern. Furthermore, to comprehensively evaluate differences among all profiles, we conducted post-estimation linear contrasts to derive odds ratios for all six possible pairwise class comparisons; the complete results are provided in **Supplementary Table S1**. All multinomial models were adjusted for gender, age, family structure, household economic situation, and parent–child relationship quality, and the overall pattern of associations between psychosocial factors and latent profile membership remained substantively unchanged when the models were estimated without these covariates.

**Table 2:** Multinomial logistic regression predicting latent profile membership.

Variable	Class	β	SE	Waldχ^2^	OR(95%CI)	P
PARS	Class2	0.026	0.002	129.097	1.026(1.022, 1.031)	0.000
	Class3	0.016	0.003	34.612	1.016(1.011, 1.022)	0.000
	Class4	0.005	0.002	6.440	1.005(1.001, 1.008)	0.011
SCSQ	Class2	0.144	0.009	234.082	1.155(1.134, 1.176)	0.000
	Class3	-0.073	0.012	35.051	0.929(0.907, 0.952)	0.000
	Class4	-0.003	0.008	0.157	0.997(0.982, 1.012)	0.692
PSSM	Class2	0.225	0.009	573.174	1.252(1.229, 1.275)	0.000
	Class3	0.161	0.008	415.330	1.175(1.157, 1.193)	0.000
	Class4	-0.007	0.006	1.638	0.993(0.982, 1.004)	0.201
CDRISC	Class2	0.296	0.013	492.020	1.344(1.310, 1.380)	0.000
	Class3	-0.048	0.010	21.684	0.953(0.934, 0.972)	0.000
	Class4	-0.016	0.008	4.529	0.984(0.969, 0.999)	0.033
SSRS	Class2	0.369	0.016	560.157	1.446(1.403, 1.491)	0.000
	Class3	0.257	0.013	402.728	1.293(1.261, 1.326)	0.000
	Class4	-0.008	0.007	1.168	0.992(0.978, 1.006)	0.280
TAI	Class2	-0.229	0.018	161.811	0.795(0.767, 0.824)	0.000
	Class3	-0.258	0.020	162.006	0.773(0.743, 0.804)	0.000
	Class4	-0.003	0.010	0.079	0.997(0.978, 1.017)	0.779

Odds ratios (OR) and 95% confidence intervals (CI) are shown for each non-reference class compared with the High Distress/High Growth–Moderate PTSD profile (Class 1). All models were adjusted for gender, age, family structure, household economic situation, and parent–child relationship quality.

(reference=High Distress/High Growth–Moderate PTSD profile, Class 1).

As shown in **Table 2**, compared to the High Distress/High Growth–Moderate PTSD profile (Class 1), adolescents in the Low Distress–High Growth profile (Class 2) were characterized by significantly higher levels of resilience (CD-RISC; OR=1.344, 95% CI [1.310, 1.380], p <.001), physical activity (PARS; OR=1.026, 95% CI [1.022, 1.031], p <.001), school belonging (PSSM; OR=1.252, 95% CI [1.229, 1.275], p <.001), adaptive coping (SCSQ; OR=1.155, 95% CI [1.134, 1.176], p <.001), and social support (SSRS; OR=1.446, 95% CI [1.403, 1.491], p <.001), along with lower trait anxiety (TAI; OR=0.795, 95% CI [0.767, 0.824], p <.001).

Pairwise contrasts from **Supplementary Table S1** further revealed several theoretically meaningful distinctions. First, physical activity and resilience emerged as key factors differentiating the two high-distress/high-growth profiles: compared to Class 1, the High Distress/High Growth–High PTSD profile (Class 4) exhibited significantly lower resilience and slightly lower physical activity. Second, when comparing the two relatively low-distress profiles, adolescents in the Low Distress–High Growth profile (Class 2) demonstrated greater resilience, physical activity, school belonging, and social support than those in the Low Growth–Moderate Distress profile (Class 3), underscoring the role of these resources in fostering higher PTG.

**Figure 4:**
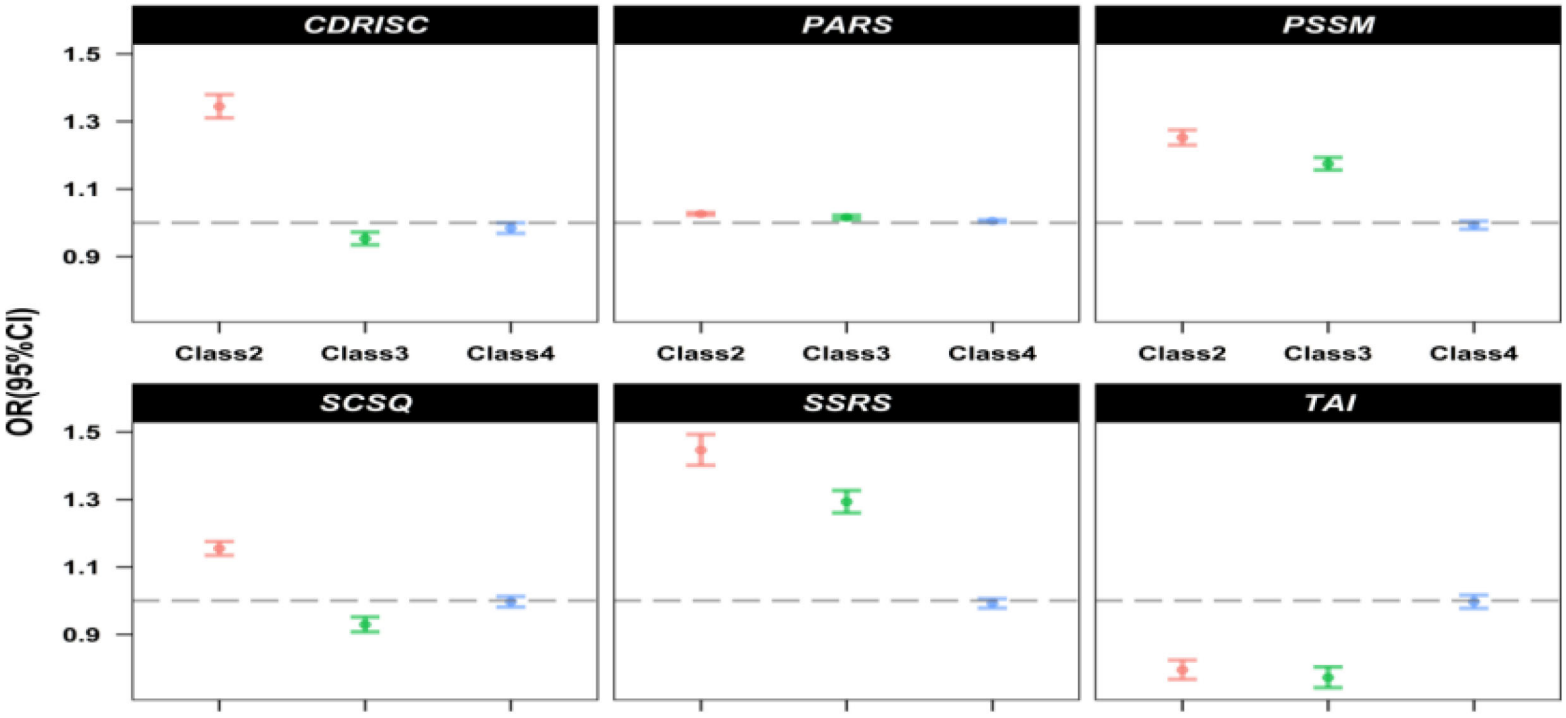
Adjusted odds ratios (95% CI) for psychosocial predictors of latent profile membership (reference=High Distress/High Growth–Moderate PTSD profile, Class 1).

These patterns are visually summarized in **Figure 4**, which plots adjusted odds ratios for each psychosocial predictor across the three non-reference classes, using Class 1 as the reference. Collectively, **Table 2**; **Figure 4** highlight that resilience, social connectedness, and physical activity were consistently associated with membership in more adaptive, growth-oriented profiles, whereas trait anxiety was consistently associated with membership in profiles characterized by more adverse trauma adaptation.

#### 3.1.5 Sensitivity analysis: correlates of high distress/high growth–high PTSD profile membership

A sensitivity analysis using binary logistic regression was conducted to identify factors specifically associated with membership in the clinically significant High Distress/High Growth–High PTSD profile (Class 4), comparing these adolescents (n=726) against all others (n=4, 318). As detailed in **Supplementary Table S4**, the results were consistent with the multinomial regression model.

After adjusting for gender, age, family structure, household economic situation, and parent–child relationship quality, three protective factors significantly reduced the likelihood of belonging to this high-distress profile: higher social support (SSRS; OR=0.965, 95% CI [0.953, 0.977], p <.001), greater school belonging (PSSM; OR=0.971, 95% CI [0.962, 0.980], p <.001), and increased resilience (CD-RISC; OR=0.974, 95% CI [0.960, 0.987], p <.001). In contrast, neither coping strategies (SCSQ) nor trait anxiety (TAI) emerged as significant predictors in this binary model (both p >.05), as shown in **Supplementary Table S4**.

These findings reinforce the central protective role of social connectedness, school belonging, and individual resilience in reducing adolescents’ risk of severe posttraumatic distress, while suggesting that coping style and trait anxiety may be less determinative for membership in this particular high-risk profile.

## 4 Discussion

Using a person-centered latent profile approach, this study identified four distinct patterns that represent co-occurring psychological states of distress and growth among Chinese adolescents in the post-pandemic context, rather than sequential stages or trajectories of adaptation. A key finding was the emergence of two mixed profiles characterized by the co-occurrence of elevated distress and substantial posttraumatic growth. This underscores the heterogeneity of adolescent responses to large-scale adversity and demonstrates that PTG can coexist with persistent symptoms of PTSD, depression, and anxiety. Regression analyses further revealed that resilience, school belonging, social support, adaptive coping, and physical activity were robustly associated with more adaptive profiles, whereas higher trait anxiety marked greater vulnerability to severe distress.

The High Distress/High Growth–High PTSD profile (Class 4), comprising 14.4% of our sample, is of particular clinical and theoretical interest. Clinically, this subgroup represents a high-risk profile that challenges conventional trauma frameworks which often posit distress and growth as opposing ends of a spectrum. The concurrent high levels of PTSD symptoms and PTG invite culturally-informed interpretation. Within the collectivist context of China, where family interdependence and social obligations are central, persistent distress may not only reflect unresolved trauma but also signify a heightened sense of collective responsibility and concern for significant others. Simultaneously, growth may be cultivated through shared meaning-making and fulfillment of familial or social roles, even amidst ongoing emotional struggle. This interpretation aligns with the dual-process model, suggesting that adolescents in this profile may engage in both intrusive and deliberate rumination, with the latter being shaped by culturally-mediated priorities such as relational harmony and filial piety.

It is crucial to note that our study design does not allow us to isolate cultural effects from other confounding factors, such as sampling frame or measurement strategies. Differences between our profiles and those reported in Western studies may stem from a combination of cultural norms, developmental stages, and methodological choices. Therefore, we interpret culture-related implications cautiously. Rather than concluding that the mixed high-distress/high-growth profile is unique to collectivist settings, we emphasize that such profiles should be understood as concurrent configurations of psychological response. Our findings generate a compelling hypothesis for future cross-cultural research: that cultural values may modulate the expression and experience of posttraumatic states, including the coexistence of distress and growth, particularly the manifestation of co-occurring distress and growth. Future studies with direct cross-cultural comparisons and measures of specific cultural values are needed to test this possibility definitively.

The pattern of psychosocial factors distinguishing the four profiles has several implications for trauma-related therapeutic and preventive efforts. First, the contrast between the two high-distress/high-growth profiles suggests that higher resilience and physical activity are particularly relevant for differentiating adolescents with comparable levels of anxiety, depression, and growth but divergent PTSD severity. This finding points to the potential value of integrating resilience-building and structured physical activity components into interventions targeting adolescents with pronounced trauma-related symptoms. Second, the comparison between the two relatively low-distress profiles indicates that greater school belonging and social support are associated with higher PTG despite similarly low levels of distress. School-based programs that foster a sense of inclusion and peer connectedness, as well as family- and community-based efforts to strengthen supportive networks, may therefore be especially helpful for promoting growth among adolescents exposed to collective adversity. Finally, elevated trait anxiety emerged as a consistent marker of vulnerability to less adaptive profiles, suggesting that early identification and anxiety-focused interventions could help prevent youth from transitioning into more severe distress trajectories.

The mixed profile characterized by high distress and relatively high growth does not necessarily indicate “unresolved trauma”; rather, it may reflect a coping pattern that is compatible with collectivist social expectations. Prior work with Chinese trauma survivors has shown that ongoing trauma narratives can coexist with reflection and positive reappraisal (18, 54, 55). In such contexts, intrusive thoughts and persistent negative affect may function not only as symptoms but also as reminders of family responsibilities and collective concerns, while growth is expressed through shared meaning-making and role fulfillment rather than solely through individualized insight. Qualitative studies similarly suggest that cultural rituals, family practices, and parental modeling can help adolescents reinterpret adversity even when distress remains elevated (56). Within a dual-process perspective, adolescents in this profile may engage in both intrusive and deliberate rumination, with moral and relational interpretation occurring alongside continued emotional struggle (57).

The pattern of psychosocial predictors further highlights the potential importance of social and institutional resources. Consistent with previous research, higher social support and school belonging were strongly associated with more adaptive profiles in our sample (58, 59). In many Chinese schools, teachers and classmates play central roles in students’ daily lives, and school communities can provide continuity and structure following collective stressors. Our findings therefore suggest that school-based programs that enhance students’ sense of belonging, together with interventions that foster resilience and encourage regular physical activity, may be useful components of trauma-informed support for adolescents. At the same time, these implications should be viewed cautiously: our study did not include a comparison group from other cultural contexts, and we did not directly measure culturally specific values or practices. Thus, we cannot attribute the observed profiles or associations solely to cultural factors. Rather, the results add to calls for more culturally pluralistic trauma frameworks (57, 58) and generate hypotheses for future cross-cultural and longitudinal research.

Several important limitations and future research directions warrant consideration. First, the cross-sectional design represents a fundamental constraint. All protective and vulnerability factors and the indicators of trauma-related distress and growth were assessed at a single time point. Consequently, the observed associations between psychosocial factors and profile membership are correlational and cannot support causal inference. The direction of effects remains ambiguous; for instance, adolescents who are already functioning more adaptively may perceive greater social support, report higher resilience, or be more likely to engage in physical activity, rather than these factors necessarily causing better adaptation. Furthermore, the multinomial logistic regression analyses relied on most-likely class assignment, which treats individuals’ most probable latent profile as known and does not propagate classification uncertainty into the regression estimates. This commonly used approach in applied LPA research may attenuate or slightly bias associations between psychosocial predictors and profile membership, so the reported odds ratios should be interpreted with appropriate caution. In addition, although we adjusted for several sociodemographic characteristics (gender, age, family structure, household economic situation, and parent–child relationship quality), these covariates do not fully capture adolescents’ broader contextual circumstances, and residual confounding by unmeasured factors (e.g., parental mental health, prior trauma exposure) cannot be ruled out. Longitudinal studies with repeated assessments—using cross-lagged panel designs or latent transition models—are essential to clarify temporal ordering, test potential causal pathways, and determine whether profiles like the High Distress/High Growth–High PTSD represent relatively stable concurrent configurations or transitional psychological states, while refraining from inferring progression or causality.

Second, given the exploratory nature of person-centered research in comparable populations, the selection of the four-profile solution and the observed prevalence of specific groups should be interpreted as preliminary. Although our sample was relatively large and drawn from multiple schools across Wuhan, it was recruited using a school-based convenience cluster strategy. Therefore, the sample should be considered school-based rather than strictly representative of all adolescents in Wuhan or other regions of China. Broader replication and cross-cultural validation using harmonized assessment instruments and analytic strategies are required to evaluate the generalizability of these profiles and to distinguish universal patterns of posttraumatic adjustment from context-specific manifestations.

Finally, several theoretical and developmental questions remain open. Future studies should directly examine the psychological mechanisms underlying the co-occurrence of distress and growth, including how culturally mediated cognitive styles (e.g., moral rumination, family-oriented self-schemas) influence trauma processing. Integrating neuroscientific approaches could further illuminate how culturally shaped developmental experiences modulate neural circuits involved in emotion regulation following trauma. Developmental perspectives are particularly needed; longitudinal research tracking different age cohorts would help clarify how interpretations of distress and trajectories of posttraumatic growth vary across adolescence and young adulthood. Addressing these gaps will significantly advance understanding of the complex interplay between cultural context, developmental stage, and trauma response.

## 5 Conclusion

This study advances our understanding of trauma adaptation among adolescents in collectivist cultural contexts, revealing four distinct profiles—Resilient/Growth, Growth-Dominant, Distressed, and the particularly noteworthy High-Risk Mixed group. The substantial presence of adolescents exhibiting concurrent posttraumatic distress and growth provides robust empirical support for dual-process models of trauma response, while underscoring the cultural mediation of emotional regulation and collective meaning-making. Contrary to traditional conceptualizations framing distress and growth as mutually exclusive, our findings suggest a culturally embedded coexistence model, wherein intrusive and deliberate rumination may operate in tandem. Within interdependent cultural frameworks emphasizing social cohesion, this duality appears not merely possible but normatively adaptive. The study extends Tedeschi and Calhoun’s posttraumatic growth theory by demonstrating how culturally salient scripts—such as filial obligation and emotional perseverance—shape adolescents’ engagement with trauma, reinforcing the need for culturally situated theoretical frameworks.

Methodologically, the application of LPA enabled a nuanced examination of trauma response heterogeneity, uncovering subgroups that conventional variable-centered approaches might obscure. This person-centered analytic strategy revealed divergent psychological trajectories following collective trauma, moving beyond aggregate-level trends to capture meaningful intra-sample variation. The proposed nested protective factor model further highlights how resilience, social support, and physical activity function not merely as individual attributes but as culturally embedded mechanisms, deeply intertwined with collectivist social structures. These findings collectively affirm that psychological adaptation is inextricably linked to its sociocultural context, necessitating trauma research and intervention paradigms that prioritize local values and relational systems. By recognizing the culturally mediated interplay between suffering and growth, this study contributes to the development of a more inclusive, globally relevant trauma science—one that balances empirical rigor with cultural sensitivity.

## Data Availability

The raw data supporting the conclusions of this article will be made available by the authors, without undue reservation.
