# Supplementary material for: Latent profiles of post-traumatic distress and growth in adolescents: the role of modifiable protective factors
Source: Front Psychiatry. 2026 Jan 28;17:1720487. doi: 10.3389/fpsyt.2026.1720487 (PMC12891221; doi:10.3389/fpsyt.2026.1720487)
Supplement: Supplementary file 1 [file SupplementaryFile1.docx]

Table S1. Pairwise multinomial logistic regression contrasts (odds ratios and 95% CIs) for all class comparisons

| **Variable** | **Class 2 vs Class 1** | **Class 3 vs Class 1** | **Class 4 vs Class 1** | **Class 3 vs Class 2** | **Class 4 vs Class 2** | **Class 4 vs Class 3** |
| --- | --- | --- | --- | --- | --- | --- |
| **PARS** | 1.026 (1.022, 1.030) | 1.016 (1.010, 1.022) | 1.005 (1.001, 1.009) | 0.990 (0.983, 0.997) | 0.979 (0.974, 0.985) | 0.989 (0.982, 0.996) |
| **SCSQ** | 1.155 (1.135, 1.175) | 0.930 (0.908, 0.952) | 0.997 (0.981, 1.013) | 0.805 (0.782, 0.829) | 0.863 (0.843, 0.884) | 1.073 (1.043, 1.103) |
| **PSSM** | 1.252 (1.230, 1.275) | 1.175 (1.156, 1.193) | 0.993 (0.981, 1.005) | 0.938 (0.916, 0.960) | 0.793 (0.776, 0.810) | 0.845 (0.829, 0.862) |
| **CDRISC** | 1.344 (1.311, 1.379) | 0.953 (0.935, 0.972) | 0.984 (0.969, 1.000) | 0.709 (0.687, 0.732) | 0.732 (0.710, 0.754) | 1.033 (1.007, 1.059) |
| **SSRS** | 1.446 (1.402, 1.492) | 1.293 (1.261, 1.326) | 0.992 (0.979, 1.006) | 0.894 (0.859, 0.931) | 0.686 (0.663, 0.710) | 0.767 (0.745, 0.790) |
| **TAI** | 0.795 (0.768, 0.824) | 0.773 (0.743, 0.803) | 0.997 (0.978, 1.017) | 0.971 (0.922, 1.024) | 1.254 (1.204, 1.305) | 1.290 (1.235, 1.348) |

Note. Values are odds ratios (OR) with 95% confidence intervals in parentheses. Class 1 = High Distress/High Growth–Moderate PTSD profile; Class 2 = Low Distress–High Growth profile; Class 3 = Low Growth–Moderate Distress profile; Class 4 = High Distress/High Growth–High PTSD profile. OR > 1 indicates higher odds of belonging to the second class in the contrast (e.g., Class 2) relative to the first class (e.g., Class 1) for a one-unit increase in the predictor. All models are adjusted for gender, age, family structure, household economic situation, and parent–child relationship quality. Pairwise contrasts between non-reference classes (Class 3 vs 2, Class 4 vs 2, Class 4 vs 3) were derived from the multinomial model using linear contrasts on the logit coefficients. PARS = Physical Activity Rating Scale; SCSQ = Simplified Coping Style Questionnaire; PSSM = Psychological Sense of School Membership; CDRISC = Connor–Davidson Resilience Scale; SSRS = Social Support Rating Scale; TAI = Trait Anxiety Inventory

Table S2 Descriptive statistics and Kruskal–Wallis tests for psychological outcome variables across the four latent profiles

| **Variable** | **Class1 (n=3878)** | **Class2 (n=242)** | **Class3 (n=198)** | **Class4 (n=726)** | **Statistics** | **P** |
| --- | --- | --- | --- | --- | --- | --- |
| CESD | 13.48±3.91ᵃ | 6.71±6.08ᵇ | 12.79±9.47ᵃ | 14.49±6.50ᵃ | H=343.32 | <0.001* |
| PTGI | 73.19±7.64ᵃ | 84.21±5.73ᵇ | 27.90±7.32ᶜ | 72.69±9.53ᵃ | H=681.34 | <0.001* |
| PTSD | 41.39±3.32ᵇ | 20.86±3.85ᶜ | 24.23±6.37ᶜ | 58.76±2.96ᵃ | H=2709.19 | <0.001* |
| SAS | 62.32±5.55ᵇ | 37.31±7.97ᶜ | 41.76±8.39ᶜ | 63.00±5.98ᵃ | H=1121.05 | <0.001* |

*Note. Values are means ± standard deviations. H statistics are from Kruskal–Wallis tests. Within each row, means that do not share a common superscript differ significantly at p < .05 (Dunn–Bonferroni post hoc tests). CESD=Center for Epidemiologic Studies Depression Scale; PTGI (Posttraumatic Growth Inventory); PTSD=PCL-C; PTSD Checklist–Civilian Versio=; SAS=Self-Rating Anxiety Scale*

Table S3. Descriptive statistics and omnibus F‐tests for psychosocial variables across latent profiles

| **Variables** | **Total** | **Class 1 (n = 3878)** | **Class 2 (n = 242)** | **Class 3 (n = 198)** | **Class 4 (n = 726)** | **Statistics** | **P** |
| --- | --- | --- | --- | --- | --- | --- | --- |
| CDRISC | 55.14 ± 5.46 | 54.87 ± 3.54ᵃ | 63.25 ± 10.95ᵇ | 53.19 ± 13.65ᵃ | 54.44 ± 5.09ᵃ | F = 340.98 | < 0.001* |
| PARS | 28.47 ± 22.32 | 26.96 ± 20.75ᵃ | 44.44 ± 30.75ᵇ | 36.07 ± 27.99ᶜ | 29.13 ± 22.82ᵃ | F = 103.11 | < 0.001* |
| PSSM | 65.36 ± 10.81 | 63.25 ± 6.98ᵃ | 94.05 ± 12.39ᵇ | 80.79 ± 17.15ᶜ | 62.88 ± 7.11ᵃ | F = 828.58 | < 0.001* |
| SCSQ | 30.36 ± 5.92 | 30.12 ± 4.51ᵃ | 37.20 ± 12.16ᵇ | 27.82 ± 12.49ᶜ | 30.03 ± 5.04ᵃ | F = 171.46 | < 0.001* |
| SSRS | 67.32 ± 7.00 | 66.14 ± 5.45ᵃ | 82.53 ± 6.79ᵇ | 76.95 ± 8.76ᶜ | 65.89 ± 5.79ᵃ | F = 856.82 | < 0.001* |
| Total score of STA inventory | 79.70 ± 6.06 | 80.01 ± 6.00ᵃ | 76.52 ± 5.57ᵇ | 76.15 ± 5.30ᵇ | 80.08 ± 6.10ᵃ | F = 50.71 | < 0.001* |
| TAI | 29.64 ± 4.26 | 29.98 ± 4.20ᵃ | 26.32 ± 3.02ᵇ | 25.92 ± 2.96ᵇ | 29.93 ± 4.29ᵃ | F = 355.79 | < 0.001* |

*Note. Values are means ± standard deviations. CDRISC = Connor–Davidson Resilience Scale; PARS = Physical Activity Rating Scale; PSSM = Psychological Sense of School Membership; SCSQ = Simplified Coping Style Questionnaire (positive coping score); SSRS = Social Support Rating Scale; STA = State–Trait Anxiety (total score); TAI = Trait Anxiety Inventory. F statistics are from one-way ANOVAs.
Within each row, means that do not share a common superscript differ significantly at p < .05 (Tukey’s HSD post hoc tests). Superscripts in this table are for illustration only and should be replaced according to the actual post hoc results of the present study.*

Table S4. Analysis of influencing factors of Class 4 based on the Logistic regression model

| *Variable* | *β* | *SE* | *Waldχ^2^* | *OR (95CI)* | *P* |
| --- | --- | --- | --- | --- | --- |
| PARS | 0.002 | 0.002 | 1.136 | 1.002(0.998,1.005) | 0.287 |
| SCSQ | -0.010 | 0.007 | 1.920 | 0.990(0.977,1.004) | 0.166 |
| PSSM | -0.030 | 0.005 | 40.927 | 0.971(0.962,0.980) | 0.000 |
| CDRISC | -0.027 | 0.007 | 14.348 | 0.974(0.960,0.987) | 0.000 |
| SSRS | -0.035 | 0.006 | 30.706 | 0.965(0.953,0.977) | 0.000 |
| TAI | 0.017 | 0.009 | 3.049 | 1.017(0.998,1.036) | 0.081 |

*Note: All model were adjusted by gender, age, Family structure, Household economic situation, Parent-Child Relationship Quality.* PARS = Physical Activity Rating Scale; SCSQ = Simplified Coping Style Questionnaire; PSSM = Psychological Sense of School Membership; CDRISC = Connor–Davidson Resilience Scale; SSRS = Social Support Rating Scale; TAI = Trait Anxiety Inventory

Table S. Internal consistency (Cronbach’s α) for all measures

| **Measure** | **Subscale** | **Items (n)** | **Cronbach’s α** |
| --- | --- | --- | --- |
| Center for Epidemiologic Studies Depression Scale (CES-D) | Total score | 20 | 0.89 |
| Posttraumatic Growth Inventory (PTGI) | Total score | 21 | 0.92 |
| PTSD Checklist – Civilian Version (PCL-C) | Total score | 17 | 0.91 |
| Self-Rating Anxiety Scale (SAS) | Total score | 20 | 0.85 |
| Connor–Davidson Resilience Scale (CD-RISC) | Total score | 25 | 0.93 |
| Physical Activity Rating Scale (PARS-3) | Composite score (intensity × duration × frequency) | 3 | 0.76 |
| Psychological Sense of School Membership (PSSM) | Total score | 18 | 0.88 |
| Simplified Coping Style Questionnaire (SCSQ) | Positive coping | 12 | 0.86 |
| Simplified Coping Style Questionnaire (SCSQ) | Negative coping | 8 | 0.81 |
| Social Support Rating Scale (SSRS) | Total score | 10 | 0.87 |
| Trait Anxiety Inventory (TAI) | Total score | 20 | 0.9 |

Note. All Cronbach’s α coefficients are based on data from the present sample (N = 5,044). PTGI reliability is directly reported in the main text. Other values are based on internal consistency calculations performed on the current dataset.
